# Inaugural Address, New York State Veterinary College

**Published:** 1896-12

**Authors:** James Law

**Affiliations:** Ithaca, N. Y.


THE JOURNAL
OF
COMPARATIVE MEDICINE AND
VETERINARY ARCHIVES.

VOL. XVII. *DECEMBER*, 1896. NO. 12.

### ADDRESS AT THE INAUGURATION OF THE NEW YORK STATE VETERINARY COLLEGE, SEPTEMBER 24, 1896.

BY PROFESSOR JAMES LAW,
ITHACA, N. Y.

(Concluded from page 781.)

But if the mere economic advantage would demand such a step, how much more would be the protection of human health and life? How much of the physical disease and death of man is due to direct transmission from corresponding disease in our domestic animals is only now beginning to be realized.

Among parasites some of the most deadly of man's tormentors come directly from our live-stock: Trichina, echinococcus, the beef and pork tapeworms, strongylus gigas, and actinomycosis may be mentioned in this connection. Among microbian diseases the list is no less redoubtable: Glanders, farcy, rabies, tetanus, milk-sickness, tuberculosis, anthrax, malignant œdema, septicæmia, erysipelas, gangrene, and infectious osteitis may be adduced as examples.

The more intimately we acquaint ourselves with the subject of communicable or contagious disease the more deeply are we impressed with the fact that there is the closest relationship and interdependence between these affections as they appear in man and animals. Indeed, in many cases, as in the echinococcus, the beef and pork tapeworms, and even the trichina, the successive appearance of man and animal as the host of the parasite at the different stages of its development is a condition of its propagation. So far as we know, it is impossible for the echinococcus or for the beef tænia to live in a host of the same genus in both its larval and mature condition. Man harbors the larva and the dog the tænia, or the calf entertains the larva and his master the tænia.

In the case of contagious affections due to microbes the same alternation from man to beast and from beast to man is not so essential to their maintenance, and yet the intimacy of the relation between the domesticated animal and the civilized man is so close that many such diseases are largely propagated in this way. In this sense glanders and anthrax stand out as largely industrial diseases. The first appear in persons having close relations to horses or to horse-products—grooms, coachmen, stablemen, cowboys, soldiers, farmers, horse-dealers, veterinarians, knackers, surgeons, tanners, gardeners—whose daily vocations lay them specially open to direct infections. The second is a disease of farmers, cattlemen, shepherds, butchers, tanners, hair- and wool-workers. But neither disease is by any means restricted to these classes. These suffer more numerously, but others suffer in a more limited extent through less direct channels of contagion. And the danger of such irregular transmission is in exact ratio with the number of diseased animals that are allowed to survive in a district. A single glandered animal is a source of no great danger. He may even be used on public highways, but his contact with or proximity to man is necessarily somewhat restricted, and the human risk is correspondingly small. But let him have free scope to infect others, and these to infect others in turn, until one can hardly enter a street without meeting an infected animal, and having him snort his deadly nasal discharge over one's person and into one's nose, eyes, and lips, and the danger at once becomes imminent. Let glanders be neglected in a street-car stable until its victims are counted by the score, or on a horse-ranch until the diseased mount up into the hundreds, and the danger, first, to the caretakers, and, second, to the general public, is greatly enhanced, and human victims of this most loathsome and deadly disease become comparatively common Let a grocer, baker, milkman, or other vendor of human food keep a glandered horse and use it in his delivery-wagon, and the hands of the driver, alternately coming in contact with the virulent discharges and the articles of food, threaten to become a very direct cause of infection to his unsuspecting customers.

A single anthrax animal would also be primarily a source of apparently little danger, but when that diseased subject is allowed to contaminate other animals, and even susceptible soil, which can retain and propagate the bacillus, the danger to both man and beast is enormously increased. Brought up from the graves by the rising of the soil-water in wet seasons, or by the intervention of the earthworm or the burrowing rodent, then dried up and blown by the winds upon the vegetation, drawn up from wells in the drinking water, borne along by streams and rivers to new localities, carried on the feet and even in the stomachs of vermin, birds, and insects, and implanted in the skin by their mandibles and biting apparatus, the bacillus finds many channels of conveyance and numerous modes of infection. Delivered from the butcher's stall into our kitchens the meat of an anthrax animal is liable to contaminate other food, through knives, forks, plates, and other articles, and even to cause direct infection through the resistance of the spore to the heat of cooking.

Of late years the general public has been more exercised over tuberculosis than any other complaint which is common to man and beast. There is doubtless good reason for this. This *white plague of the North*, by far the most deadly affection of man, killing one-eighth of civilized humanity, and attacking one-fourth or even one-third at some period of their lives, is also the most prevalent chronic disease of our dairy herds; and its extension in the human race bears a remarkable ratio to the utilization of the bovine races for dairy products and beef. Piscivorous tribes, like the western islanders of Scotland, are usually remarkably free from tuberculosis, as are also the native Chinese, who are vegetarians. The ruling Tartar race in China, on the other hand, are beef-eaters and largely tuberculous. In Egypt and Algiers, in the comparative absence of bovine herds, the great influx of consumptives has not materially deteriorated the health of the native population; while in Italy, Australia, Hawaii, and Madeira, where the population freely consume the products of the bovine race, the rush of phthisical health-seekers has led to a great extension of tuberculosis among the natives. Among tribes of our own Indians who feed on the raw flesh of the ox, too often diseased, 50 per cent. of the total mortality is from tuberculosis.

Concurrent testimony obtained on so large a scale and from such widely different sources is not to be lightly set aside.

Tuberculosis is mostly a chronic disease, frequently lasting through a long lifetime. A certain number of cases recover; many more remain dormant, ready to burst into renewed activity whenever the health is otherwise undermined. It is essentially a "pestilence that walketh in darkness," and often under an outward guise of health the subject of the disease carries around the germs of death to his unsuspecting and more susceptible fellow. The very latency of the disease in certain systems, and the absence of all prominent outward manifestations of illness, are potent factors in the propagation of the infection. A disease that is quickly fatal, like smallpox, plague, yellow fever, or cholera in man, or anthrax, rinderpest, or Texas-fever in cattle, is easily dealt with, since, wherever the germ exists in connection with susceptible subjects, its presence will be speedily manifested, and it can easily be circumscribed and crushed out. These make their attack in broad daylight, as with great sound of trumpet and roll of drum, and we are warned to fortify every pass and strengthen every defence. But the stealthy tubercle bacillus, which glides up in the darkness and silence, and as it were, saps our walls of defence without visible manifestation or audible sound, and suddenly appears, when least expected, in the interior of our most trusted keep, is by far the most dangerous enemy.

To neglect our defence because of this subtlety is to abandon our cause and play the poltroon. This is not the part of modern science; this is not the course of the medicine of to-day and of the future. To the biologist who has studied the infinitesimal forms of parasitic life, the subtlety of the germ is but a challenge to meet its inroads by a more effective strategy, to meet its hidden mines by equally able countermines, and to turn an otherwise assured defeat into an accomplished victory. But in doing this he can never safely abandon the first principles of warfare. He can never avoid a favorable opportunity to reduce the numbers of the enemy, nor to prevent him from securing reinforcements. Yet this is just the course that is strongly urged in regard to tuberculosis. Because some recover, and because other cases remain long latent, we are urged to let such cases go on in their work of indefinitely multiplying the disease-germ, and to attack them only when they become acute and deadly. Acute cases do not live long to propagate the disease. Whence then comes the constant succession of cases? Mainly from the latent and recovering ones.

Our own university herd is a standing example of a sound prevention of this infection. Formerly affected with tuberculosis, it has now for a number of years been entirely free from the affection, in spite of many risks, from visitors and otherwise, and in spite even of the presence in the barn, for several months, of a latent and unsuspected case, which had been brought from another herd. Had we left that dormant case in the herd after its discovery, it would in all probability have sooner or later developed into active disease, and become the source of a new general extension of tuberculosis in the herd.

It is impossible in a short lecture to lay down infallible and iron rules for dealing with this or any similar disease under all possible circumstances. Special conditions may warrant special measures. In the case of valuable animals where economic considerations would warrant the supervision, separate herds of dormant cases may be allowed for breeding purposes, if they can be kept carefully apart from all other stock, their milk products denied to man or animals, and all acute cases weeded out from the herd as soon as they can be detected. Above all, if such breeding-herd of dormant cases can be subjected to a continuous out-of-door life on the open prairie, where the chances for recovery are highest and the risks of contagion lowest, they may be made profitable by fattening their healthy progeny for beef, or still more so by the perpetuation of a valuable strain of blood. Under such professional supervision and frequent testing the actually recovered animals could in due time be removed from their still questionable companions and restored to a guaranteed herd.

But the one who would argue from this that the actual, though somewhat latent and dormant, cases should be left in the herd that has been tested and proved to be above suspicion, is but pleading for a free field for the propagation of the contagion. The acute cases that would develop at intervals would entail new victims, no longer among the latent cases and suspected animals only, but among the tested and sound as well.

Under average conditions, with low-priced cattle and a State indemnity, the slaughter of all the tuberculous would be the course of economical and successful sanitary work; and when special condition rendered another and less radical resort permissible, it should only be adopted when hedged about by such precautions as would obviate danger to man and beast.

We know enough about the dreaded tuberculosis to say that we can deal with it successfully under the most varied conditions; but our past achievement does not imply that we have as yet reached the limit of possible success in this disease; and a similar success for a whole State or nation would not warrant us in saying that no better measures can be taken. Such a conclusion would be utterly unscientific and unduly conservative. It is the best at present known to us; but in these days, when knowledge advances by leaps and bounds, no one can say what to-morrow may have in store for us. Some as-yet-unknown Edison or Tesla may be even now preparing a surprise in the revelation and utilization of natural forces of which we little dream, and which may cast into the shade our steam engines, our electric telegraphs, telephones, phonographs, and skiagraphs.

So in the field of biology and modern medicine. The largest hopes and the brightest ideas are likely to prove the most scientific. The vivid imagination and the scientific foresight must unite to help in our future progress. Not in the case of tuberculosis alone, but in connection with the entire field of medicine, a whole phalanx of possibilities, big with promise for the future of humanity, confront us.

As biologists, we see genera, species, and even varieties of animals that are largely insusceptible to this and that deadly disease. It is for us to grasp the cause of this immunity, and, if possible, to render it available over a wider area.

As bacteriologists, we recognize incompatibilities and antagonisms between the living cells of the animal body and their products on the one hand, and the pathogenic microbe and its products on the other. How far can we avail of these to strike a balance favorably for and protective to the animal? We are as yet on the mere confines of this great science of bacteriology. In the vast microscopic world, full of attractions and repulsions of living cells and microbes, of neutralizations and physiological antagonisms of leucomaïnes, ptomaïnes, toxins, and enzymes, of sozins and phylaxins, there are many and bright promises for the future of preventive and therapeutic medicine. But it is only the trained mind, rich with the knowledge already attained in this science, that can hope to achieve the triumphs of the future. Knowledge, skill, imagination, sound judgment, and indefatigable industry must combine in the man who would hope for success in this field. It is no place for the dull or the laggard.

Without undue arrogance it may be asserted that to us has been allotted a large measure of responsibility in relation to this work. By the generosity of the Empire State we are enabled to enter on the field. We have been furnished with the nucleus of a scientific institution from which large and important results may fairly be expected. We are honored as being in a sense the pioneers in a comparatively new field; we have the place of advanced guard in the inevitable warfare. Though small in numbers, our chosen battlefield is one in which numbers count for less than quality, and in respect to quality we have to prove ourselves. Let us take as our primary thought the Socratic aphorism, "Knowledge is virtue; ignorance is crime." In our case this is preëminently—I may say, painfully—true. As the beneficiaries of the State we shall prove unthankful and unworthy if we fail to make the best use possible of its bounty. As trusted representatives of science it is expected of us that we fortify ourselves with the lore of the past, and strike out with clear vision, steady foot, and strong hand for future achievement. To rest satisfied with any knowledge short of the best of to-day is to neglect our opportunity and prove untrue to our trust. The lore of the past can never be safely set aside nor entirely ignored, yet this has led up to so much that is more recent, clearer, more definite, and full of so much greater potency, that, with Socrates, we may say it is criminal to neglect even its smallest lessons. The accumulated knowledge of the ages is great and indispensable, but is small indeed unless we build upon it the riper fruits of its own modern development. This is true for teacher and student alike; for are we not all students in one common school? Some of us may have advanced to a higher grade, while some are but entering the lowest class, but success will crown each only as he devotes his best energies to his work in the spirit of truth and with the ardor of the enthusiast. In this as in all else we must approve ourselves as men. The veterinary profession has long suffered from the low appreciation in which it has been held. Everyone who has conceived an attachment for animals has thought himself competent to deal with their diseases. Our State is crowded with men who without further preparation or fitness have been legally established as veterinarians by a simple registration of their names as such. To the future graduate it is given to redeem the profession from this low public estimate. He must everywhere approve himself, first, as a man of character, a good man, and a good citizen; next, he must approve himself as a man of science. His judgment and his word must be authoritative on all matters that involve his profession and the great interests connected with animal industry. He must be an educator in the highest sense. Wherever his lot may be cast, with whatever class of domestic animals he may be called upon to deal, he must charge himself with the task of bringing to the work the accumulated knowledge of centuries, and especially of the wonderful century which is drawing to its close.

Some of you may be called upon to engage in the extinction of animal plagues. In this spotless integrity must be joined to the highest knowledge and skill, and conjoined to a deep insight into human nature and an inflexible purpose of applying even-handed justice, if you would escape the danger of being overwhelmed in the storm of detraction and misrepresentation that will inevitably assail you. The honorable prize to be won is great one, but it requires a good soldier and a sterling man to bear the brunt. When the complete triumph comes you will find that your whilom detractors, who have opposed you in good faith, will come forward to acknowledge their error and indorse your achievement.

Some will be called to inspect markets and food products; and here, with the weighty responsibility of a city's health on your shoulders, you will bless the day that brought you through the rigorous studies of anatomy, physiology, histology, pathology, toxicology, and enabled you with scientific certainty to indorse the wholesome and condemn the diseased and unwholesome.

Some, I trust, will be called to fill the chairs of comparative medicine, now for the first time being established, in the most forward medical schools, and which must soon be provided in all such schools that are worthy of the name. None can fill such a place so well as the man who has profoundly studied the special diseases of animals, and, indeed, none other is fitted to do justice to such a chair. Ever since Hippocrates the most advanced physicians have recognized and employed the lower animals as a means of advancing medical knowledge. Our knowledge of physiology largely consists in deductions from experiments made upon the lower animals; our acquaintance with the physiological action of drugs and poisons very largely consists in accurate observations made upon the lower animals; pathology, surgery, and medicine owe much—very much—to the same source; and bacteriology is essentially based upon experimentation on the beast. Comparative medicine, therefore, can be best pursued by the veterinarian, who, otherwise equally well furnished with the medical candidate, adds to his accomplishments a thorough practical knowledge of all animal diseases. This will quicken his insight into pathogenic causes and the significance of morbid phenomena, will protect him against hasty and erroneous conclusions, and will make his work at once more productive and more reliable.

Others will be called to undertake investigations in our agricultural experiment stations, where the same wide and accurate knowledge, the same keen insight and skill, and the same scientific methods can alone bring out valuable results.

For all such fields of usefulness you must make ample and thorough preparation. Patient labor, earnest and systematic effort, daily accomplishment of the day's problems in a thorough manner, will make the work easy and assure success. In entering this institution you begin in a very special sense your work of life. Outside the professional school the work of preparation has been essentially general and introductory. In the professional college you start upon what you have especially chosen as your life's work. We who are somewhat older in the field are appointed to advise and guide you in the preliminary stages. It is our purpose and hope to do our whole duty by you in the right spirit. We bespeak your earnest effort to do your whole duty by the subject in hand, so that the foundations, at the laying of which we mutually labor, may grow up into a grand, noble, and worthy development, an honor to your alma mater and to our benefactor, the State of New York.

